# A temporal basis for Weber's law in value perception

**DOI:** 10.3389/fnint.2014.00079

**Published:** 2014-10-14

**Authors:** Vijay Mohan K. Namboodiri, Stefan Mihalas, Marshall G. Hussain Shuler

**Affiliations:** ^1^Department of Neuroscience, Johns Hopkins UniversityBaltimore, MD, USA; ^2^Allen Institute for Brain ScienceSeattle, WA, USA

**Keywords:** Weber's law, scalar timing, subjective value, reward, uncertainty, decision making

## Abstract

Weber's law—the observation that the ability to perceive changes in magnitudes of stimuli is proportional to the magnitude—is a widely observed psychophysical phenomenon. It is also believed to underlie the perception of reward magnitudes and the passage of time. Since many ecological theories state that animals attempt to maximize reward rates, errors in the perception of reward magnitudes and delays must affect decision-making. Using an ecological theory of decision-making (TIMERR), we analyze the effect of multiple sources of noise (sensory noise, time estimation noise, and integration noise) on reward magnitude and subjective value perception. We show that the precision of reward magnitude perception is correlated with the precision of time perception and that Weber's law in time estimation can lead to Weber's law in value perception. The strength of this correlation is predicted to depend on the reward history of the animal. Subsequently, we show that sensory integration noise (either alone or in combination with time estimation noise) also leads to Weber's law in reward magnitude perception in an accumulator model, if it has balanced Poisson feedback. We then demonstrate that the noise in subjective value of a delayed reward, due to the combined effect of noise in both the perception of reward magnitude and delay, also abides by Weber's law. Thus, in our theory we prove analytically that the perception of reward magnitude, time, and subjective value change all approximately obey Weber's law.

## Introduction

Weber's law, or approximate Weber's law, has been observed in the perception of stimulus features such as weight (Weber, 1978; Killeen et al., 1993), length (Dehaene and Brannon, 2011; Droit-Volet, 2013; Akre and Johnsen, 2014), brightness (Rovamo et al., 1995), number (Whalen et al., 1999; Cordes et al., 2001; Nieder and Miller, 2003; Cantlon and Brannon, 2006; Beran, 2007; Gallistel, 2011; Droit-Volet, 2013), reward magnitude (Killeen et al., 1993; Bateson et al., 1995; Kacelnik and Bateson, 1996), time (Gibbon, 1977; Gibbon et al., 1984; Matell and Meck, 2000; Buhusi and Meck, 2005), loudness (Forrest, 1994; Bee et al., 2012) etc. (Akre and Johnsen, 2014). It states that the ability to perceive a change in a quantity decreases in proportion to its magnitude. The fact that our ability to perceive a change in a stimulus often decreases as its magnitude increases is immediately recognized; for instance, it is more difficult to perceive an increase of 1 g if one is measuring 100 g as opposed to when measuring 2 g. Weber's law, however, states that this decrease in ability to assess magnitude is proportional to the magnitude of the stimulus, i.e., that it is 50 times more difficult to perceive a given change around 100 g than it is to perceive the same change around 2 g. Even though there is considerable experimental support for the law, its neural or evolutionary origin is unclear (Walsh, 2003; Bueti and Walsh, 2009; Akre and Johnsen, 2014). Further, since animals are often thought to make decisions so as to maximize reward rates (thus requiring perception of reward magnitude and delays) (Stephens and Krebs, 1986; Balci et al., 2011; Blanchard et al., 2013; Namboodiri et al., 2014b), Weber's law in the perception of reward magnitudes and delays must affect such decisions. The mathematical properties of such effects on the decisions of animals are, however, unclear.

Previously, we presented a theory of decision-making and time perception that postulates that the decision of animals regarding delayed outcomes is a consequence of reward rate maximization in a limited temporal window that includes a past integration interval (over which experienced reward rate is estimated) and the delay to a given reward (TIMERR) (Namboodiri et al., 2014b). We showed that the decision-making algorithm resulting from this postulate automatically includes an estimate of opportunity cost and an explicit cost of time. We further showed that it can explain the breadth of behavioral observations on intertemporal decision-making. The theory also postulates that time is represented subjectively such that the subjective reward rate equals the objective change in reward rate, i.e., a subject's estimate of the subjective value per unit subjective time accurately represents how much the reward rate of the current offer exceeds the experienced reward rate. Using this theory, we examine the origin of Weber's law in reward magnitude in this paper and show that the perception of reward magnitude is correlated with the perception of time, and that the subjective value change of a delayed reward should also approximately abide by Weber's law. We also present a novel accumulator model of sensory perception that predicts approximate Weber's law for quantities (such as reward magnitude) that are measured over finite sensory intervals.

## Results

Our main aim in this paper is to study how errors in the subjective representation of an interval correspondingly affect the subjective value of that reward. To this end, we first express the subjective value of a delayed reward in terms of the subjective representation of the delay.

**Figure 1:**
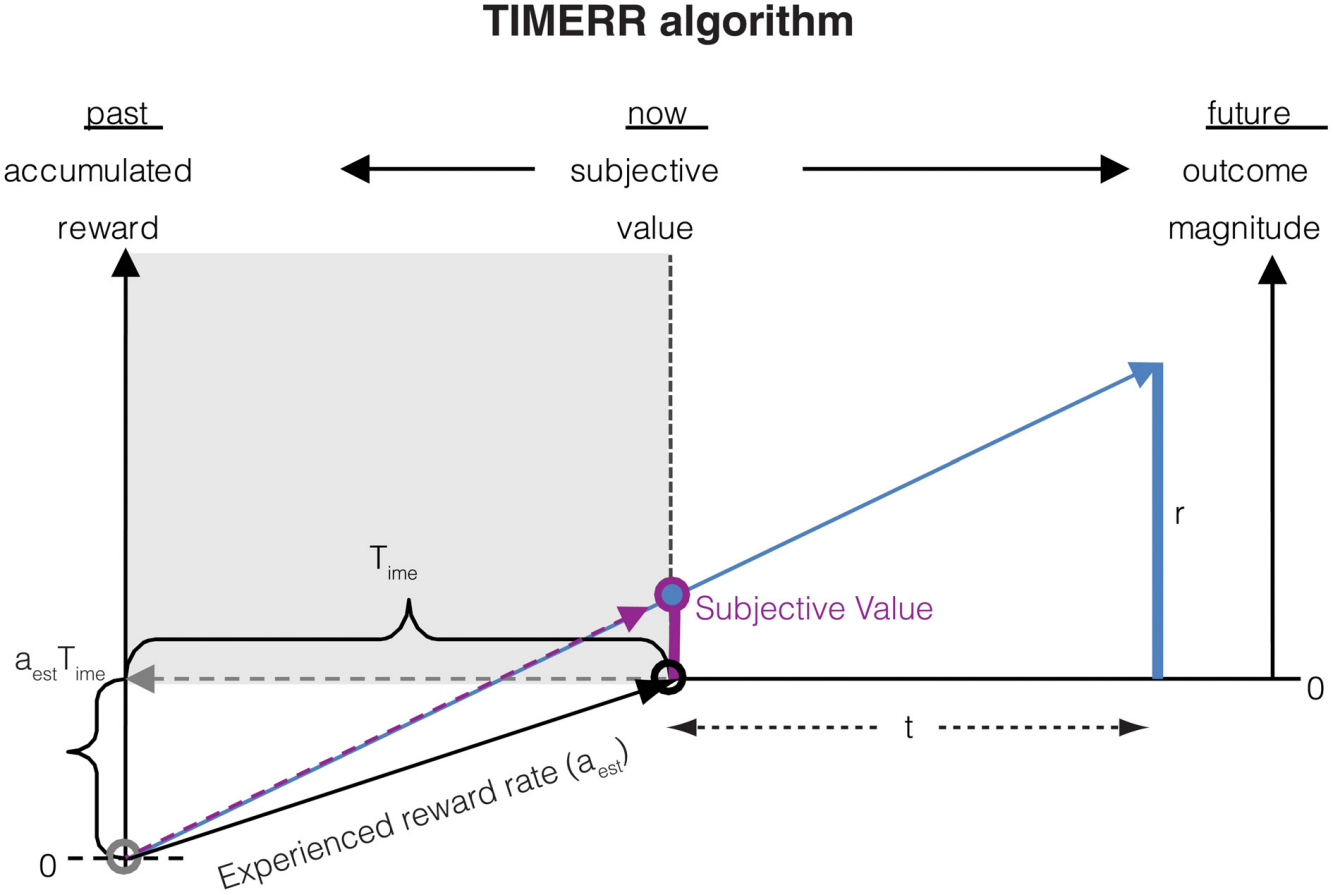
**The subjective value of a delayed reward (*r*) is calculated as the immediate reward that produces the same total reward rate over a window including a past-integration interval (*T_ime_*) (over which experienced reward rate is calculated, *a_est_*) and the expected delay (*t*) to a future reward**. The figure shows a ready means to visually depict the subjective value of a delayed reward, shown as the purple bar at time zero (“now”). Modified from Namboodiri et al. (2014a).

The subjective value of a reward with magnitude *r* delayed by an interval *t* as calculated in TIMERR (Figure 1) is:

(1) $$SV\left(r,t\right)=\frac{r-a_{est}t}{1+\frac{t}{T_{ime}}}$$

where *T_ime_* represents the past integration interval, i.e., the interval over which the past reward rate (*a_est_*) is estimated. Importantly, *T_ime_* is not a perceived temporal interval, but is merely the effective interval over which the past reward rate is estimated (e.g., using an exponential memory filter as in Namboodiri et al., 2014a).

In the above equation, *r* can be thought of as the magnitude of an offered reward. But a more rigorous definition of *r* is the subjective value of an immediate offered reward, i.e., *r* = *SV*(*r*, 0).

Correspondingly, the subjective representation of the delay *t* as expressed in TIMERR is:

(2) $$ST\left(t\right)=\frac{t}{1+\frac{t}{T_{ime}}}$$

Thus, the subjective representation of time is a non-linear mapping and its non-linearity is controlled by the past integration interval. It is important to emphasize that the subjective representation of the delay expressed above is not the subjective (verbal) report of an interval; it can be thought of as the non-linear neural representation of an interval.

Equation (1) can now be re-expressed in terms of the subjective representation of time as shown in Equation (2) as

(3) $$\begin{array}{l} SV\left(r,t\right)=\frac{r}{1+\frac{t}{T_{ime}}}-a_{est}ST(t)=r\frac{ST(t)}{t}-a_{est}ST(t)\\ \;=r\frac{ST(t)}{\frac{ST(t)}{1-\frac{ST(t)}{T_{ime}}}}-a_{est}ST(t) \end{array}$$

Therefore,

(4) $$SV\left(r,t\right)=r-\left(\frac{r}{T_{ime}}+a_{est}\right)ST(t)$$

Thus, the discounting of a delayed reward is linear with respect to the subjective representation of that delay. We assume here that the subjective value of a delayed reward is calculated by first measuring the subjective representation of the delay and then linearly discounting using the form expressed in Equation (4). This linear discounting with respect to the subjective representation of time is a direct result of the postulate of our theory that animals maximize reward-rates over a limited temporal window including the past integration interval and the delay to future reward.

### Contribution of time measurement error to the error in subjective value

**Figure 2:**
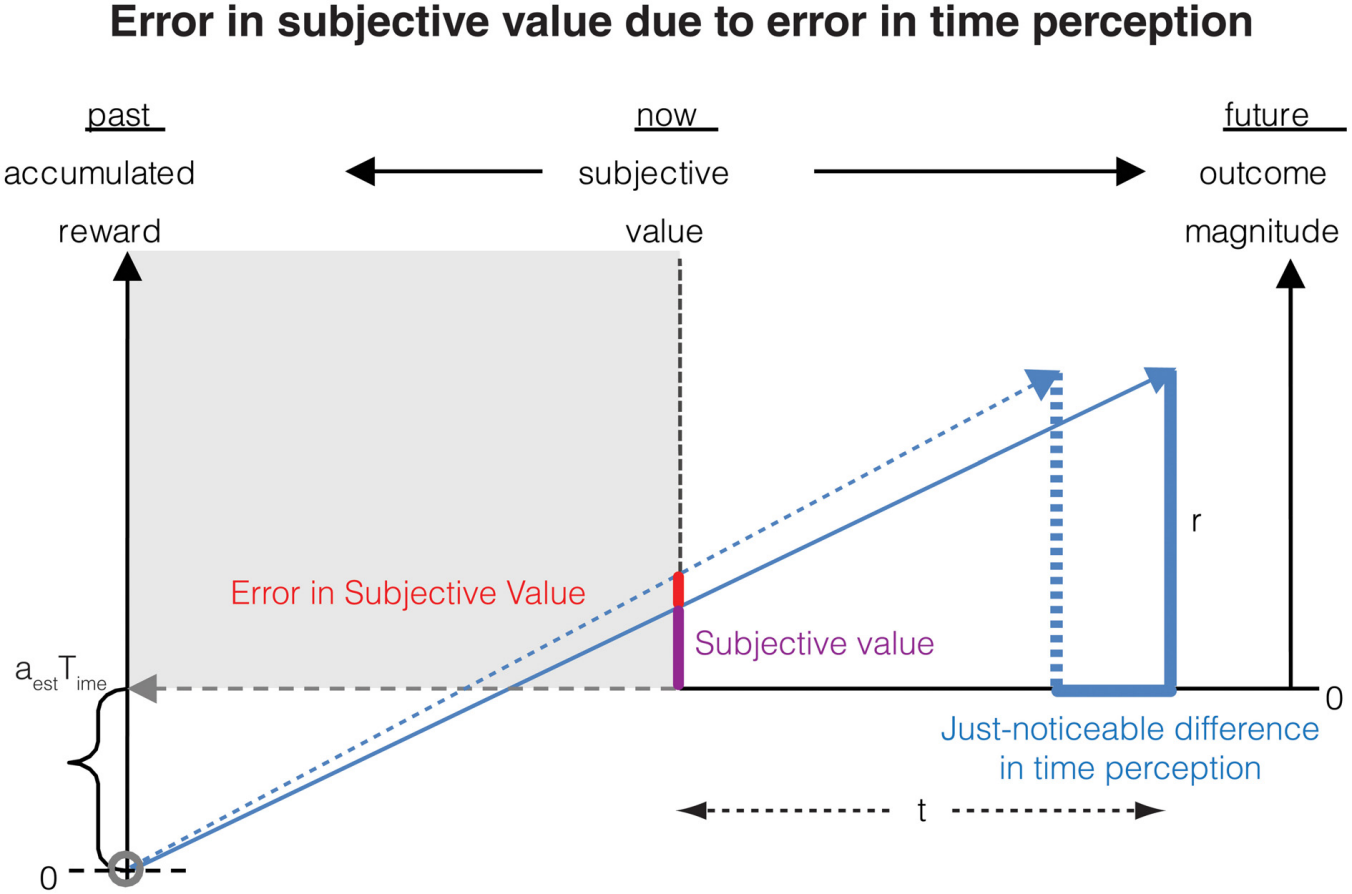
**Errors in measurement of the delay to a future reward results in a corresponding error in subjective value**. If the delay to the reward is perceived as earlier by the just-noticeable difference (JND), the subjective value is perceived as being larger. This error in subjective value is shown in the red bar and is calculated analytically in Section Contribution of time measurement error to the error in subjective value.

From this relation, we can now calculate the error in subjective value of a delayed reward resulting from an error in the representation of subjective time (Figure 2). To this end, let us denote that the just-noticeable-difference (JND) in the subjective representation *ST(t)* of the delay *t* by δ*ST(t)*, and that the error in the corresponding subjective value is denoted by δ*SV*(*r, t*). For the purpose of this section, we assume that the measurement of the reward magnitude is noiseless. Then, as the subjective representation of the delay *t* increases by its JND, the subjective value will increase by the corresponding error. This can be expressed mathematically as:

(5) $$SV(r,t)+\delta SV(r,t)=r-\left(\frac{r}{T_{ime}}+a_{est}\right)(ST(t)+\delta ST(t))$$

From Equations (4, 5), we can now calculate the JND in subjective value as

(6) $$\delta SV(r,t)=-\left(\frac{r}{T_{ime}}+a_{est}\right)\delta ST(t)$$

The negative sign here implies that as the delay increases, the subjective value decreases, i.e., the value is discounted.

We have previously shown that the error in the subjective representation of time is approximately linearly related to the subjective representation of time in an accumulator model (Namboodiri et al., 2014b), i.e., δ*ST(t)* = *kST(t) + c*. The contribution due to the constant term *c* can be thought of a constant read-out error and is quite small except in the limit of *ST(t)* approaching zero. Substituting this relationship into Equation (6), we get

(7) $$\delta SV(r,t)=-\left(\frac{r}{T_{ime}}+a_{est}\right)(kST(t)+c)$$

Equation (7) can also be rewritten using Equation (4) as

(8) $$\delta SV(r,t)=-c\left(\frac{r}{T_{ime}}+a_{est}\right)-k(r-SV(r,t))$$

From the above equation, it can be seen that the error in subjective value of a delayed reward is linearly related to the drop in subjective value from time zero due to the passage of time. Hence, Weber's law applies for the reduction in subjective value of a delayed reward due to the delay, i.e., to *r* − *SV*(*r, t*). In other words, as the delay increases and the subjective value reduces, the error in the change of subjective value due to the delay is proportional to the change in subjective value. Henceforth, we refer to this as Weber's law in value perception.

Let us now examine the effect of reducing the delay to zero. Since the negative sign in the above equations only indicates the direction of change, we drop this sign from here on for the calculation of noise. Thus, when *t* = 0, both Equations (7, 8) become

(9) $$\delta SV(r,0)=c\left(\frac{r}{T_{ime}}+a_{est}\right)$$

**Figure 3:**
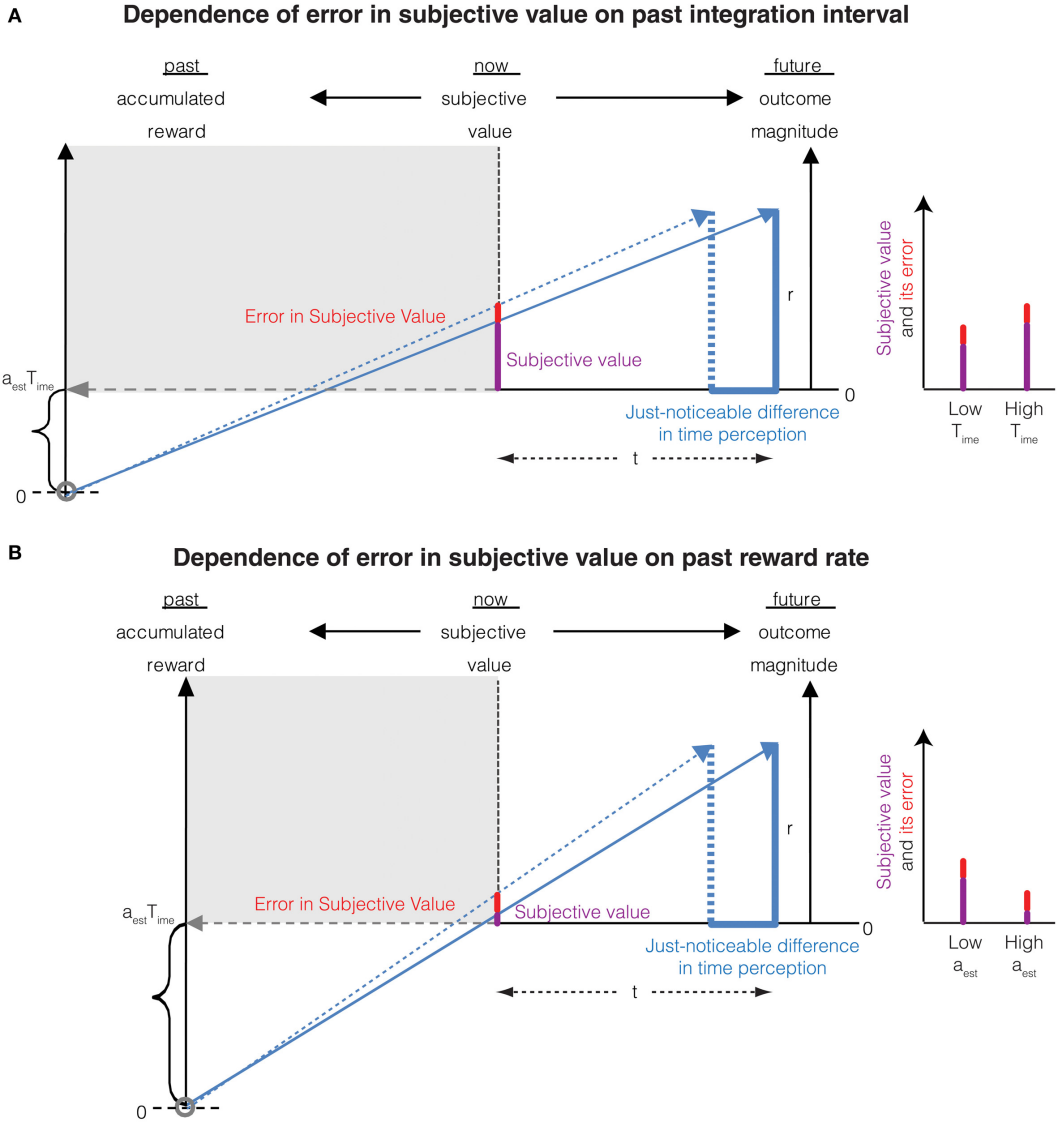
**(A)** The Weber fraction of error in subjective value decreases with an increase in the past integration interval. This is mathematically represented in Equation (8). Compared to Figure 2 (represented as low *T_ime_* in the graph on the right), the past integration interval is larger in this panel, thus reducing the error in subjective value while increasing the subjective value. The Weber fraction is thus smaller. **(B)** The Weber fraction of error in subjective value increases with an increase in the past reward rate. This is mathematically represented in Equation (8). Compared to Figure 2 (represented as low *a_est_* in the graph on the right), the past reward rate is larger in this panel, thus increasing the error in subjective value while decreasing the subjective value. The Weber fraction is thus larger.

Thus, the error in the subjective value of an immediate reward is proportional to the magnitude of the reward. This is Weber's law in magnitude perception resulting purely from an error in the perception of an infinitesimally small immediate delay rather than arising solely from magnitude measurement error as is commonly believed. Interestingly, as the past integration interval (*T_ime_*) increases—leading to an increased accuracy of time perception (Namboodiri et al., 2014b)—so does the accuracy of reward magnitude perception. This is a novel, untested prediction of the account presented here. This temporal basis of Weber's law for the perception of reward magnitude also predicts that the accuracy of magnitude representation reduces when the past reward rate is high. This too, is a novel, testable prediction and is consistent with the notion that when reward rate is high, the need to represent rewards accurately (thus incurring greater metabolic costs) is reduced. The above two predictions regarding the dependence of errors in subjective value on the past integration interval and the past reward rate is depicted in Figure 3.

The above treatment indicates that noise in time perception results in reward magnitude perception that abides by Weber's law. Yet note that, to calculate the error in subjective magnitude (resulting purely from the noise in the measurement of the infinitesimally-small delay to immediate reward), we heretofore have ignored the contribution of noise in the measurement of the reward magnitude itself. Since we do not know the relative contributions of these sources of noise, it is possible that the source related to time might contribute but minimally to the overall error in reward magnitude perception. Therefore, in the next section, we present a model of sensory perception for reward magnitude, and then calculate the resulting perceptual error.

### Sensory measurement error of reward magnitude due to evidence accumulation

In this section, we calculate the sensory measurement error of reward magnitude (e.g., error in the measurement of the volume of a water reward). In some modalities, the sensory receptor is itself thought to produce scalar noise (Matthews et al., 1990; Donner et al., 1998; Nieder and Miller, 2003). While this is possible in the measurement of reward magnitude, we do not consider this simple solution here as neural elements in the central nervous system are typically considered to approximate Poisson processes, which have square-root noise and not linear noise (Rieke et al., 1999). Rather, we consider errors in ascribing value to a given reward magnitude as resulting from central and not peripheral processes. While there are other models for Weber's law in sensation (Treisman, 1966; Dehaene, 2003; Deco and Rolls, 2006; Shouval et al., 2013), ours is based on the fact that the measurement of any sensory quantity has to be carried out over time.

To this end, we assume that the sensory process for measuring the magnitude is carried out in time over a small temporal window of sensation. This sensory window is defined as the time over which there is a constant rate of sensory input. Hence, we assume that the net perceived reward magnitude is proportional to the time it takes to integrate the sensory input (e.g., when drinking water at a constant rate, the amount of water obtained is proportional to the duration of consumption). For an alternative model of sensory integration, see Appendix A1 in Supplementary Material. In order to evaluate the noise in measurement, we assume that this sensory integration can be described by an accumulator model similar to previous decision-making models used for evidence accumulation (e.g., Simen et al., 2011; Brunton et al., 2013). We further assume that the reward magnitude is represented linearly and does not undergo a logarithmic transformation, as has been suggested for number representation (Dehaene, 2003). In the rest of this section, we formalize this accumulator model using a stochastic differential equation, and then analytically calculate the time dependence of its mean and variance.

If the neural system carrying out this sensory integration were perfectly noiseless, we can describe the accumulator model by the following differential equation

(10) $$dr_{t}=adt;\;0\leq t\leq t_{sensory}$$

Here, *r_t_* represents the integrated reward magnitude at a given time. Thus, the measured reward magnitude *r* will be the integrated magnitude at the end of the sensory window, *t_sensory_*, i.e., *r* = *r_t_sensory__*. The rate of sensory input is denoted by *a*.

We now relax the assumption that the sensory integration is noiseless. Noise in such an accumulator system can result from two sources: noise in the sensory input and feedback noise in the accumulator. We assume that the feedback is a zero mean noise resulting from balanced excitatory/inhibitory connections, similar to many previous works (e.g., Simen et al., 2011; Brunton et al., 2013), and that the neurons performing these computations can all be described as Poisson point processes, i.e., the variance of each source of noise will be proportional to the corresponding signal. Thus, the variance of the sensory input will be proportional to the input (*a*) and the feedback noise will be proportional to *r_t_*. We denote the proportionality constants as *b* and σ respectively.

For simplicity, we first assume that these two sources of noise are independent and additive. Since the variance of the sum of two independent sources sum up, the net variance can be expressed as σ^2^*r*_*t*_ + *b*^2^*a*. If we consider the variance of the noise term as constant throughout the integration, it can be represented by introducing an additional diffusive term that approximates a Brownian motion with infinitesimal variance of σ^2^*r*_*t*_ + *b*^2^*a* into Equation (10). Thus, the introduction of these noise sources can be formally described by the following stochastic differential equation

(11) $$dr_{t}=adt+\sqrt{\sigma^{2}r_{t}+b^{2}a}\;dW_{t};\;0\leq t\leq t_{sensory}$$

*W_t_* represents a standard Wiener process (Brownian motion).

We will analytically solve the time dependence for the first and second moments of the above accumulator [shown in Equation (11)] so as to calculate the mean and variance at the end of the sensory window.

Taking the expectation values on both sides of Equation (11), we get

(12) $$d<r_{t}>\;=\;adt;\;0\leq t\leq t_{sensory}$$

where < *r*_*t*_ > represents the expectation value of *r*_*t*_. Since < *r*_0_ > = 0, we can write the solution obtained by integrating from 0 to *t* as

(13) $$<r_{t}>=at$$

The time evolution equation for < *r*^2^_*t*_ > can similarly be calculated by applying Ito's product rule as

(14) $$\begin{array}{l} dr_{t}^{2}=2r_{t}dr_{t}+{\left(dr_{t}\right)}^{2}=2ar_{t}dt+2r_{t}\sqrt{\sigma^{2}r_{t}+b^{2}a}\;dW_{t}\\ \;+{\left(adt+\sqrt{\sigma^{2}r_{t}+b^{2}a}\;dW_{t}\right)}^{2}0\leq t\leq t_{sensory} \end{array}$$

Using *dt*^2^ = 0, *dW*_*t*_*dt* = 0, and *dW*^2^_*t*_ = *dt* and taking the expectations of both sides, we get

(15) $$\begin{array}{l} d<r_{t}^{2}>=\left(2<ar_{t}>+<\sqrt{\sigma^{2}r_{t}+b^{2}a}\;.\;\sqrt{\sigma^{2}r_{t}+b^{2}a}>\right)dt;\\ \;0\leq t\leq t_{sensory} \end{array}$$

Simplifying, we get

(16) $$d<r_{t}^{2}>=\left((\sigma^{2}+\text{2}a)<r_{t}>+b^{2}a\right)dt;\;0\leq t\leq t_{sensory}$$

Substituting from Equation (13) and integrating from 0 to *t* with the boundary condition of < *r*^2^_*t* = 0_ > = 0, we get

(17) $$<r_{t}^{2}>=a(\sigma^{2}+2a)\frac{t^{2}}{2}+b^{2}at;0\leq t\leq t_{sensory}$$

Thus, the variance of *r*_*t*_ can be calculated as

(18) $$\text{var}(r_{t})=<r_{t}^{2}>-<r_{t}{>}^{2}=\frac{a\sigma^{2}t^{2}}{2}+b^{2}at;\;0\text{​}\leq t\text{​}\leq t_{sensory}$$

The coefficient of variation of *r*_*t*_ is thus

(19) $$CV(r_{t})=\sqrt{\frac{\sigma^{2}}{2a}+\frac{b^{2}}{at}};\;0<t\leq t_{sensory}$$

Since the measured reward magnitude is the integrated magnitude after the sensory window, the CV of the measurement can be written as

(20) $$CV(r)=\sqrt{\frac{\sigma^{2}}{2a}+\frac{b^{2}}{at_{sensory}}}$$

or

(21) $$CV(r)=\sqrt{\frac{\sigma^{2}}{2a}+\frac{b^{2}}{r}}$$

**Figure 4:**
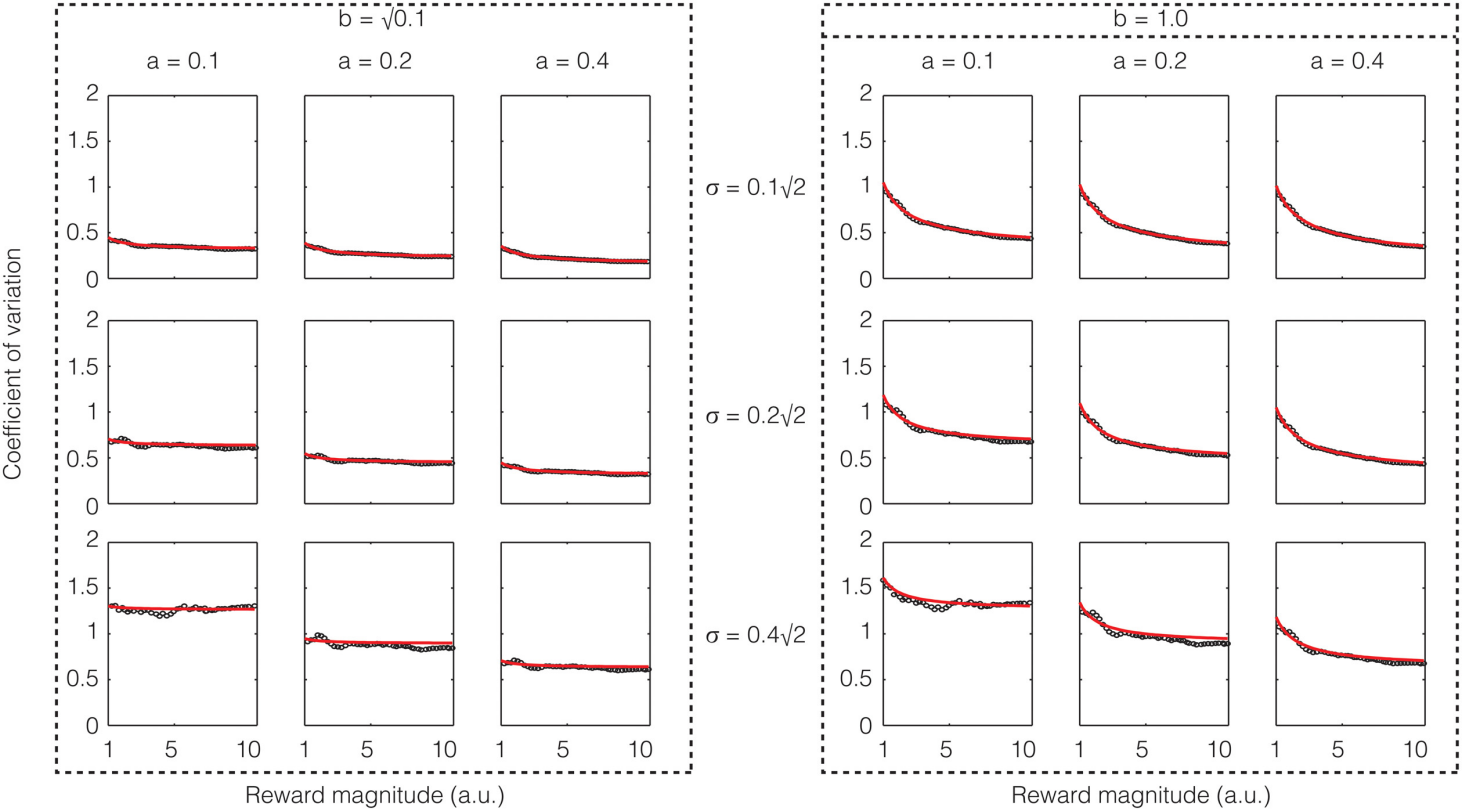
**Confirmatory simulations (see Methods) of the analytical solution of an accumulator model in which the sensory and feedback noise combine additively**. The red line shows the result of the analytical calculation as expressed in Equation (21), wherein the sensory signal (*a*), magnitude of sensory noise (*b*), and the magnitude of feedback noise (σ) are varied. The black dots show the results of numerical simulation. The results approximate Weber's law well but for low reward magnitudes and high sensory noise (*b*).

If one assumes that the rate of sensory input is a constant, the above equation shows that except for low reward magnitudes, the CV is a constant, i.e., Weber's law holds approximately for reward magnitude perception. If σ^2^/*a* is large compared to *b*^2^, the constant term will dominate and the CV would be almost exactly constant. These analytical results are confirmed in numerical simulations as shown in Figure 4.

The mathematics of the accumulator shown in Equation (11) is quite similar to Equation (9) in (Simen et al., 2011). But there are some significant differences in the meaning of the terms. First, our model is for reward magnitude perception, whereas theirs is for time interval production. Second, as a consequence, while in our model the rate of sensory input is assumed to be a constant, they assume that the rate of accumulation is tuned for the interval to be timed. For this reason, their model can produce scalar timing only for time interval production and not for time interval measurement/perception where the coefficient of variation decreases in inverse proportion to the square root of the interval [similar to the second term in Equation (21)].

Equations (11–21) assumed that the sensory input noise is additive with respect to the feedback noise. Instead, if this noise were in fact multiplicative, Equation (11) would change to

(22) $$dr_{t}=adt+\sigma \sqrt{b^{2}ar_{t}}\;dW_{t};\;0\leq t\leq t_{sensory}$$

In this case, the CV can similarly be calculated as (shown in Appendix A2 in Supplementary Material)

(23) $$CV(r)=\frac{\sigma b}{\sqrt{2}}$$

Thus, when the sensory and feedback noises multiply, the coefficient of variation is independent of the magnitude of the sensory signal (*a*).

**Figure 5:**
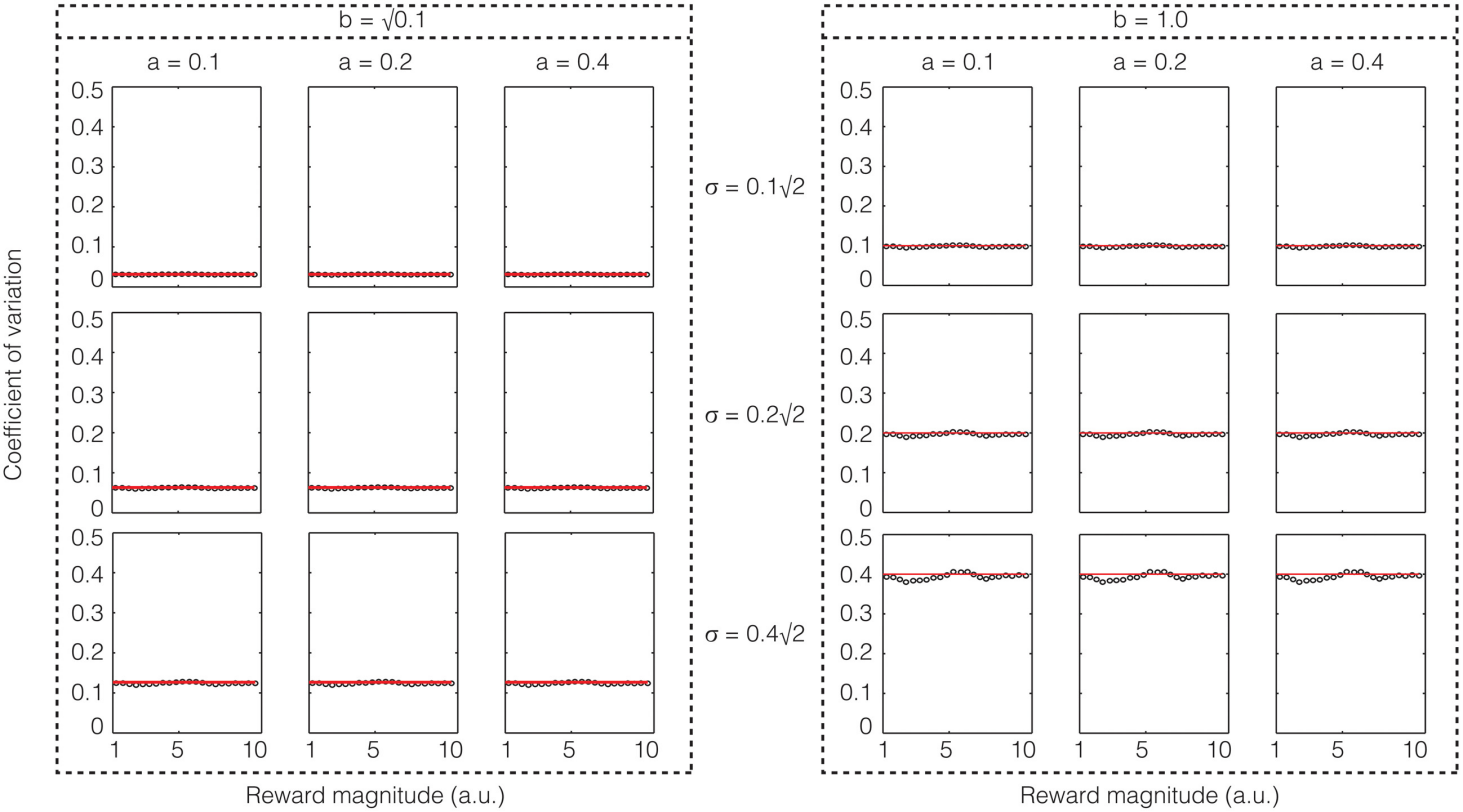
**Confirmatory simulations (see Methods) of the analytical solution of an accumulator model in which the sensory and feedback noise combine multiplicatively**. The red line shows the result of the analytical calculation as expressed in Equation (23) wherein the sensory signal (*a*), magnitude of sensory noise (*b*), and the magnitude of feedback noise (σ) are varied. The black dots show the results of numerical simulation. Here, Weber's law is exact.

Again, we performed confirmatory numerical simulations of Equation (22), the results of which are shown in Figure 5. Therefore, if the sensory input noise is multiplicative, the coefficient of variation is exactly constant, thus making Weber's law exact. Instead, if the sensory input noise is additive, the coefficient of variation shows deviations from exact Weber's law at low reward magnitudes.

The accumulator model considered above is similar to the one that we previously proposed for the representation of subjective time (Namboodiri et al., 2014b), with two differences. The most important difference is that whereas subjective time is assumed to be a non-linear transform of real time, subjective reward is assumed to be linearly proportional to the real reward. Due to this difference, the reward magnitude accumulator is analytically tractable, unlike the subjective time accumulator, for which the analytical solution was approximate (Namboodiri et al., 2014b). The other difference is that since the reward magnitude accumulator operates on a sensory input (unlike the subjective time accumulator), the contribution of this sensory noise has also been included.

### Combined error due to time and magnitude measurements on subjective value

**Figure 6:**
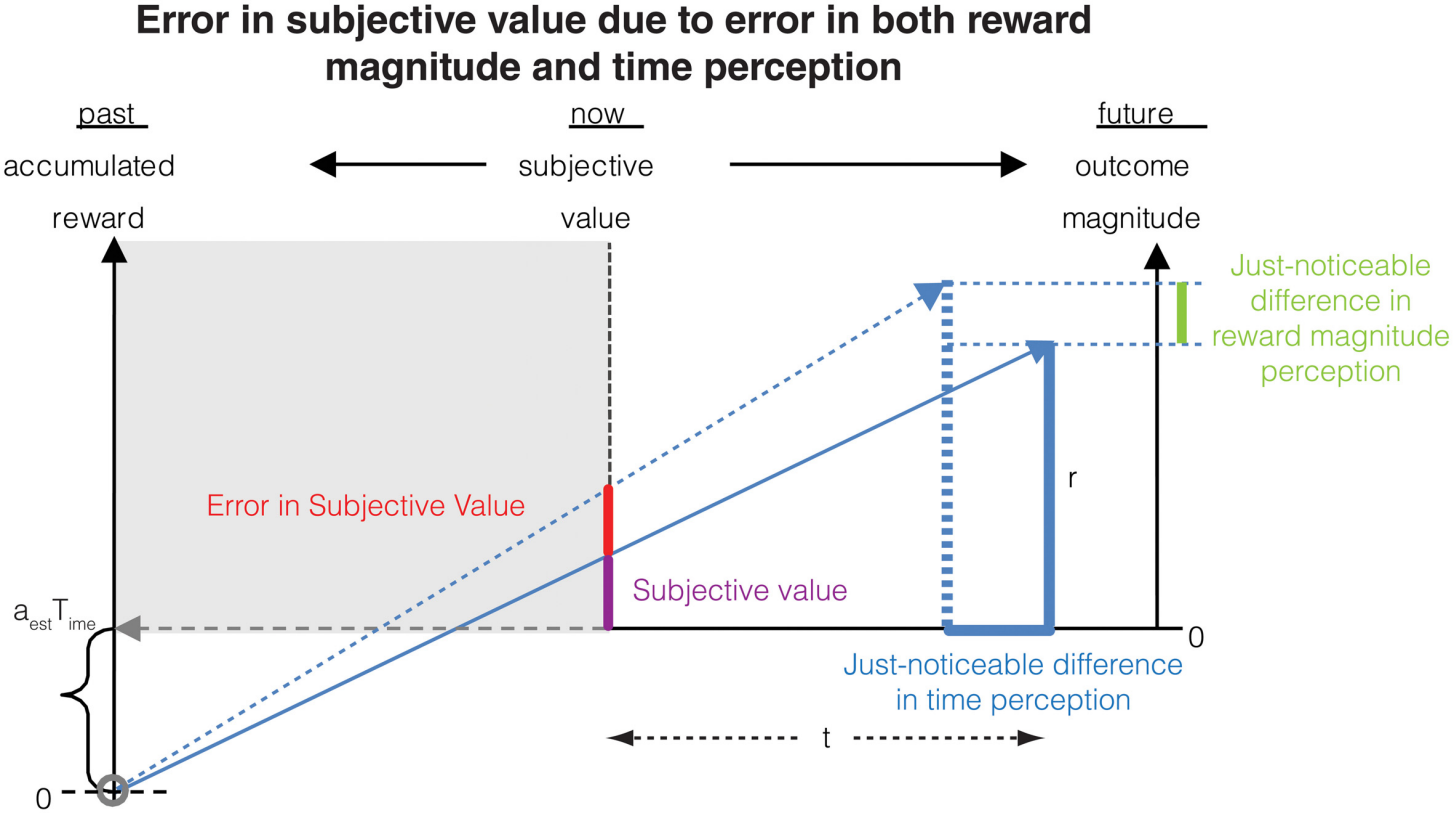
**The error in subjective value is affected by errors in the measurement of both delay (as shown in Figure 2) and reward magnitude**. This combined error is calculated analytically in Section Combined error due to time and magnitude measurements on subjective value.

We now have all the elements to calculate the error in subjective value of a delayed reward resulting from errors in both magnitude and time measurements (Figure 6).

Returning to Equation (4), if we consider the effect of adding the JND of both *r* and *ST(t)*, we see that while adding the JND of *r* leads to an increase in the *SV*(*r, t*), adding the JND of *ST(t)* leads to a decrease (due to temporal discounting). Since we are only interested in the net error, so as to match the direction of change, we will consider the effect of error in both *r* and *ST(t)* by adding the JND of *r* and subtracting the JND of *ST(t)*. Thus, we get the following equation

(24) $$\begin{array}{l} SV(r,t)+\delta SV(r,t)=r+\delta r-\left(\frac{r+\delta r}{T_{ime}}+a_{est}\right)(ST(t)\\ \;-\delta ST(t)) \end{array}$$

Therefore, using Equation (4), the error in subjective value δ*SV*(*r, t*) can be written as

(25) $$\begin{array}{l} \delta SV(r,t)=\delta r\left(1-\frac{ST(t)}{T_{ime}}\right)+\left(\frac{r}{T_{ime}}+a_{est}\right)\delta ST(t)\\ \;+\;\frac{\delta r\delta ST(t)}{T_{ime}} \end{array}$$

From Equation (4), $\left(1-\frac{ST(t)}{T_{ime}}\right)=\frac{SV(r,t)+a_{est}ST(t)}{r}$. Therefore, Equation (25) becomes

(26) $$\begin{array}{l} \delta SV(r,t)=\frac{\delta r}{r}\left(SV(r,t)+a_{est}ST(t)\right)+\left(\frac{r}{T_{ime}}+a_{est}\right)\delta ST(t)\\ \;+\;\frac{\delta r\delta ST(t)}{T_{ime}} \end{array}$$

For simplicity, we consider the exact form of Weber's law to hold for the sensory measurement of *r*. Therefore, we write $\frac{\delta r}{r}=l$, where *l* is the Weber fraction.

From Equations (6), (8), the second term in the R.H.S is equal to $c\left(\frac{r}{T_{ime}}+a_{est}\right)+k(r-SV(r,t))$, where δ*ST*(*t*) = *kST*(*t*) + *c*.

Before calculating the error in subjective value at any delay, we first calculate its value for an immediate reward, where *t* = 0 and *ST*(*t*) = 0. From Equation (26), this can be written as

(27) $$\delta SV(r,0)=lr+c\left(\frac{r}{T_{ime}}+a_{est}\right)+\frac{lcr}{T_{ime}}$$

Simplifying, we get

(28) $$\delta SV(r,0)=r\left(l\left(1+\frac{c}{T_{ime}}\right)+\frac{c}{T_{ime}}\right)+ca_{est}$$

The above equation obeys Weber's law for reward magnitude perception, resulting from errors in both the measurement of magnitude and the measurement of the infinitesimal delay to an immediate reward. As can be seen, the Weber fraction [slope of δ*SV(r, 0)* with respect to *r*] depends on *T_ime_*, the past integration interval. Thus, we predict that even within an individual, the Weber fraction in the perception of reward magnitude (subjective value of an immediate reward) can change depending on the context, as the past integration interval changes. The direction of this change will be such that the better the perception of time, the better the perception of reward magnitude. Further, as mentioned previously after Equation (9), the above equation also predicts that the larger the experienced reward rate, the larger the error in perception of reward magnitude. These are the strong falsifiable predictions of our account.

We now calculate the error in subjective value at a given delay *t* due to errors in both time and reward magnitude measurement. From Equation (26), we get

(29) $$\begin{array}{l} \delta SV(r,t)=l\left(SV(r,t)+a_{est}ST(t)\right)+c\left(\frac{r}{T_{ime}}+a_{est}\right)\\ \;+\;k(r-SV(r,t))+\frac{lr(kST(t)+c)}{T_{ime}} \end{array}$$

Simplifying, we get

(30) $$\begin{array}{l} \delta SV(r,t)=(l-k)SV(r,t)+\left(la_{est}+\frac{lrk}{T_{ime}}\right)ST(t)\\ \;+\;r\left(\frac{(1+l)c}{T_{ime}}+k\right)+ca_{est} \end{array}$$

Since we are interested in the noise in subjective value of a constant reward magnitude delayed by varying amounts, if we treat *r* as a constant (for now), we can write [using Equation (4)] $ST(t)=\frac{r-SV(r,t)}{\frac{r}{T_{ime}}\;+\;a_{est}}$. Grouping the terms that are proportional to *SV*(*r, t*) separately from the other terms, the above equation becomes

(31) $$\begin{array}{l} \delta SV(r,t)=\left(l-k-l\frac{a_{est}T_{ime}+rk}{a_{est}T_{ime}+r}\right)SV(r,t)\\ \;+\left(r\left(\frac{(1+l)c}{T_{ime}}+k+l\frac{a_{est}T_{ime}+rk}{a_{est}T_{ime}+r}\right)+ca_{est}\right) \end{array}$$

The above equation also abides by Weber's law. Thus, we have shown that the error in subjective value of a given reward delayed by different amounts is proportional to the subjective value at each given delay. Again, the Weber fraction depends on the reward environment of the animal since it depends on *r*, *a*_*est*_, and *T_ime_*.

We can also similarly calculate the subjective value error at a given delay for differing reward magnitudes. To do this, we substitute *r* as [using Equation (4)] $r=\frac{SV(r,t)+a_{est}ST(t)}{1-\frac{ST(t)}{T_{ime}}}$ in a rewritten version of Equation (25) as shown below.

(32) $$\begin{array}{l} \delta SV(r,t)=lr\left(1-\frac{ST(t)}{T_{ime}}\right)+c\left(\frac{r}{T_{ime}}+a_{est}\right)\\ \;+\;k\left(\frac{r}{T_{ime}}+a_{est}\right)ST(t)+\frac{lr(kST(t)+c)}{T_{ime}} \end{array}$$

Thus,

(33) $$\begin{array}{l} \delta SV(r,t)=l\left(SV(r,t)+a_{est}ST(t)\right)+r(1+l)\frac{c+kST(t)}{T_{ime}}\\ \;+\;a_{est}(c+kST(t)) \end{array}$$

Or,

(34) $$\begin{array}{l} \delta SV(r,t)=SV(r,t)\left(l+(1+l)\frac{c+kST(t)}{T_{ime}-ST(t)}\right)+a_{est}(lST(t))\\ \;+\;ST(t)(1+l)\frac{c+kST(t)}{T_{ime}-ST(t)}+c+kST(t)) \end{array}$$

where $ST(t)=\frac{t}{1+\frac{t}{T_{ime}}}$.

This too abides by Weber's law. Thus, we have also shown that the error in subjective value at a given delay for different reward magnitudes is proportional to the subjective value.

## Discussion

Previously, we presented a general theory of intertemporal decision-making and time perception (TIMERR) that explains many well-established observations in these fields (Namboodiri et al., 2014b). Our theory states that the decisions of animals are a consequence of maximizing reward rates in a limited temporal window including a past integration interval and the delay to a current reward. Interestingly, we showed that the representation of time is also related to the past integration interval in our framework, and that impulsive (low tolerance to delays of rewards) individuals have an impaired perception of time. We then demonstrated that the error in perception of time is approximately scalar, with the deviation from exact Weber's law depending on the past integration interval.

In this paper, we extend the results of our prior work to consider the role of error in time perception on the perception of reward magnitudes and the subjective values of delayed rewards. We showed that the error in perception of the infinitesimally small delay to an immediate reward affects the perception of reward magnitude in accordance with Weber's law. Since the sensory measurement of the reward must be carried out over time, we derived Weber's law in the sensation of reward magnitude by assuming an accumulator model (for this sensory integration) with a Poisson feedback with balanced excitation/inhibition. This could be the underlying reason behind the observation of Weber's law in the perception of reward magnitude by animals. Subsequently, we showed that in TIMERR, the combination of errors in both time and reward magnitude measurement on the subjective value change of a delayed reward also accords with Weber's law. Crucially, the Weber fractions are predicted to depend on the reward history of the animal, thus providing a strong, falsifiable prediction of our theory, along with the predicted correlation between errors in time perception and reward magnitude estimation.

Superficially, it might be assumed that since the perception of reward magnitude abides by Weber's law, so should the subjective value of a delayed reward. In fact, such an assertion has previously been made (Cui, 2011) without the recognition that this requires a specific relation between subjective value, reward magnitude, delay to reward, and the perception of the delay. From our analytical derivation presented above, it is evident that Weber's law in subjective value change is a consequence of the special forms of discounting function (subjective value of a delayed reward divided by the subjective value of that reward when presented immediately) and subjective time representation that result from our theory. In fact, if one were to make the standard assumptions of (1) Weber's law in reward magnitude measurement, (2) a hyperbolic discounting function (Ainslie, 1974; Frederick et al., 2002; Kalenscher and Pennartz, 2008; Cui, 2011), and (3) linear subjective representation of time that abides by Weber's law (Gibbon, 1977; Gibbon et al., 1984), the resultant error in subjective value of a delayed reward is far from proportional to the subjective value, as we show in Appendix A3 in in Supplementary Material.

Recent experiments have shown that the representation of reward magnitude or value is not just dependent on the reward under consideration, but also on other available options (Huber et al., 1982; Bateson et al., 2003; Louie et al., 2013). A recent neuroeconomic model (Louie et al., 2013) employing a divisive normalization scheme wherein each individual reward is compared against the other available options can produce such context dependence. In light of these findings, one might question our assumption of an absolute code for reward magnitude, i.e., our assumption that reward magnitude is represented based only on the magnitude of the reward of interest. It is thus important to point out that our theory predicts context dependent choices even under the assumption that the reward magnitude representation is independent of the other available options. This is because the subjective value of a reward (since every reward is effectively a delayed reward) is affected by the animal's estimate of its past reward rate [Equation (1)]. Thus, the presence of distracters affects the subjective value of a reward due to an effect on the past reward rate in experiments involving sequential choices. Additionally, the current options might affect one's estimate of experienced reward rate (Namboodiri et al., 2014a). Further, as shown in Equations (9, 28), the larger the value of the past reward rate, the larger the error (Weber fraction) in representation of a reward. Thus, our theory predicts that the larger the value of the distracter (thereby making the past reward rate larger), the higher the errors in deciding between two rewards, in accordance with the experimental observations shown in Louie et al. (2013). The key difference between our account and the divisive normalization account (Louie et al., 2013) is that in our account, the context dependence is due to the estimation of past reward rate, whereas in divisive normalization, the context dependence is based only on the currently available options.

There have been prior models of how Weber's law in reward magnitude and time perception affects decisions of animals in the context of external variability along these two dimensions (see Kacelnik and Bateson, 1996; Kacelnik and Brito e Abreu, 1998). These models have been successful at explaining why animals tend to prefer variability in time, but not in reward magnitude, in comparison with fixed options of the same mean (see Kacelnik and Bateson, 1996; Kacelnik and Brito e Abreu, 1998). However, they do not propose an origin of Weber's law for reward magnitude or time, nor do they calculate the net error due to both sources of noise. Unique to our theory, we predict a systematic relationship between the reward history of animals and their perception of these quantities.

## Methods

The confirmatory simulations performed for Figures 4, 5 integrated Equations (11, 22) respectively using the Euler-Maruyama method. Thus, the discrete time version of the equation used for Figure 4 was

(35) $$r_{t+\Delta t}=r_{t}+a\Delta t+\sqrt{\sigma^{2}r_{t}+b^{2}a}\sqrt{\Delta t}N(0,1)$$

and that for Figure 5 was

(36) $$r_{t+\Delta t}=r_{t}+a\Delta t+\sigma b\sqrt{ar_{t}}\sqrt{\Delta t}N(0,1)$$

where *N(0,1)* is the standard normal distribution. The step size for integration, *Δt*, was set to 0.001 units. The parameters were changed as shown in the figure legend. In each case, the same random seed was used to initialize the simulations.

### Conflict of interest statement

The authors declare that the research was conducted in the absence of any commercial or financial relationships that could be construed as a potential conflict of interest.
