# Supplementary material for: A temporal basis for Weber's law in value perception
Source: Front Integr Neurosci. 2014 Oct 14;8:79. doi: 10.3389/fnint.2014.00079 (PMC4196632; doi:10.3389/fnint.2014.00079)
Supplement: Supplementary file 1 [file DataSheet1.DOCX]

# Appendix

## A1

In the paper, we assumed that the sensory integration for the measurement of reward magnitude is carried out at a constant rate and that the net perceived magnitude is proportional to the time of integration. Instead, it is possible to assume that the sensory integration is carried out over a constant window and that the rate of accumulation is proportional to the reward magnitude. This may be more appropriate when the reward magnitude depends on the quality of the reward (juice vs. water) instead of the quantity (more water vs. less water). In this case, under the assumption of additive independent Poisson noise as used for the derivation of Equation , the coefficient of variation in the integration would be exactly as shown in Equation , with the only difference being that the perceived magnitude *r* would depend on *a* and that *t_sensosry_* would be a constant. Hence, in this case, the coefficient of variation would be inversely proportional to the square root of the reward magnitude, thus not obeying Weber’s law.

If instead the noises are multiplicative as assumed in Equation , the coefficient of variation will indeed be a constant as shown in Equation , independent of the rate of accumulation. Thus, we see that if the sensory and feedback noise were multiplicative, Weber’s law would be exact.

## A2

Here we derive the coefficient of variation for the case of multiplicative noise as expressed in Equation (reproduced below).

$$dr_{t}=adt+\sigma\sqrt{b^{2}ar_{t}}\text{ }dW_{t};\text{ }0\leq t\leq t_{sensory}$$

Taking the expectation values on both sides, we get

$$d<r_{t}>\text{ }=\text{ }adt;\text{ }0\leq t\leq t_{sensory}$$

where <*r_t_*> represents the expectation value of *r_t_*. Since <*r_0_*>=0, we can write the solution obtained by integrating from 0 to *t* as

$$<r_{t}>\text{ }=\text{ }at$$

The time evolution equation for <*r_t_^2^*> can be calculated by applying Ito’s product rule

$$dr_{t}^{2}\text{ }=\text{ 2}r_{t}dr_{t}\text{+(}dr_{t}\text{)}^{2}\text{=2}ar_{t}dt+2r_{t}\sqrt{\sigma^{2}r_{t}b^{2}a}\text{ }dW_{t}\text{+(}adt+\sqrt{\sigma^{2}r_{t}b^{2}a}\text{ }dW_{t}\text{)}^{2}\text{; }0\leq t\leq t_{sensory}$$

Using *dt^2^=0, dW_t_ dt=0* and *dW_t_^2^=dt* and taking the expectations of both sides, we get

$$d<r_{t}^{2}>\text{ }=\text{ (2<}ar_{t}>+<\sqrt{\sigma^{2}r_{t}b^{2}a}.\sqrt{\sigma^{2}r_{t}b^{2}a}>)dt;\text{ }0\leq t\leq t_{sensory}$$

Simplifying, we get

$$d<r_{t}^{2}>\text{ }=\text{ (}\sigma^{2}b^{2}a+2a)<r_{t}>dt;\text{ }0\leq t\leq t_{sensory}$$

Substituting <*r_t_>=at* and integrating from 0 to *t* with the boundary condition of <*r_t=0_^2^*>=0, we get

$$<r_{t}^{2}>\text{ }=\text{ }a^{2}\text{(}\sigma^{2}b^{2}+2)\frac{t^{2}}{2};\text{ }0\leq t\leq t_{sensory}$$

Thus, the variance of *r_t_* can be calculated as

$$\mathrm{var}(r_{t}\text{) =}<r_{t}^{2}>-<r_{t}>^{2}=\text{ }\frac{a^{2}\sigma^{2}b^{2}t^{2}}{2};\text{ }0\leq t\leq t_{sensory}$$

The coefficient of variation of *r_t_* is thus

$$CV=\frac{b\sigma}{\sqrt{2}}$$

**A3**

Here we calculate the effect of the noise in the measurement of reward magnitude and delay on the subjective value of a delayed reward that is discounted purely hyperbolically, with the time perception being linear and showing Weber’s law in error. This subjective value can be written as

$$SV(r,t)=\frac{r}{1+kt}$$

Since this is not a polynomial function, the JND in *SV(r,t)* will have to be approximated using a Taylor series.

If we only consider, for now, error in time measurement, the JND for *SV(r,t)* can be written as

$$\delta SV(r,t)=-\frac{kr}{(1+kt)^{2}}\delta t+\frac{2k^{2}r}{(1+kt)^{3}}\frac{\delta t^{2}}{2}+....$$

Keeping only the first order term, we get

$$\delta SV(r,t)\approx-k\frac{SV(r,t)^{2}}{r}\delta t$$

Assuming Weber’s law in time perception, we can write $\delta t=ct$. Substituting into the above equation, we get

$$\delta SV(r,t)\approx-kc\frac{SV(r,t)^{2}}{r}\frac{(r-SV(r,t))}{kSV(r,t)}$$

Simplifying, we get

$$\delta SV(r,t)\approx\left|-c\frac{SV(r,t)}{r}(r-SV(r,t))\right|=cSV(r,t)-c\frac{SV(r,t)^{2}}{r}$$

where the negative sign has been ignored since we are only interested in the magnitude of the noise. Thus, the error in subjective value of a hyperbolically-discounted reward in the presence of scalar timing is quadratic with respect to the subjective value, to the first order.

If we now also include the error in measurement of reward magnitude, the first order term for *δSV(r,t)* can be written as

$$\delta SV(r,t)=\frac{\delta r}{1+kt}+\frac{kr}{(1+kt)^{2}}\delta t$$

Here, the JND in time perception was decreased so as to maintain the same direction of change as increasing the reward magnitude by its JND. If *δr=lr*, this can be written as

$$\delta SV(r,t)=lSV(r,t)+cSV(r,t)-c\frac{SV(r,t)^{2}}{r}$$

This is also quadratic with respect to the subjective value to the first order approximation.

Therefore, even if Weber’s law held in reward magnitude and time perception, under the common belief of hyperbolic discounting, the subjective value of a delayed reward does not abide by Weber’s law.
